# Can inhibiting insulin/IGF signaling with dietary carbohydrate restriction play a role in treatment/prevention of cancers?

**DOI:** 10.1186/2049-3002-2-S1-O31

**Published:** 2014-05-28

**Authors:** Eugene J Fine, C Segal-Isaacson, Silvia Herzkopf, Joseph Sparano, Maria Romano, Richard Feinman, Nora Tomuta, Amanda Bontempo, Abdissa Negassa

**Affiliations:** 1 Nuclear Medicine, Albert Einstein College of Medicine, Bronx, NY, USA; 2 Radiation Oncology, Montefiore Medical Center, Bronx, NY, USA; 3 Medicine, Montefiore Medical Center, Bronx, NY; 4 Cell Biology, SUNY-Downstate Medical Center, Brooklyn, NY, USA; 5 Epidemiology and Public Health, Albert Einstein College of Medicine, Bronx, NY, USA

## Background

Hyperinsulinemia, hyperglycemia, and obesity have been identified as risk factors for a variety of cancers [1]. Insulin inhibition (INSINH) can potentially limit cancer growth by factors including ketosis [2], and apoptosis secondary to fatty acid synthase inhibition [3] as well as intracellular potassium depletion [4]. Furthermore, dysregulation of many signaling proteins downstream of the insulin/IGF receptors such as PI3K/Akt, mTOR (inhibition) and AMPK (amplification) is a major area of drug target development [5,6]. We performed a four week INSINH diet in patients with advanced cancers to study safety/feasibility and also to examine a change in^18^F-2-fluoro, 2-deoxyglucose (FDG) uptake on PET scan as a surrogate measure for tumor response.

## Methods

Eligible patients had failed or refused ≥2 standard chemotherapy courses. Exclusions included concurrent chemotherapy, end-organ disease, hypoglycemic medications, difficult compliance, or BMI < 20. A supervised INSINH diet restricted starches and sugars for 28 days, and was monitored weekly for macronutrient intake, body weight, [glucose] [BHB], [insulin], [IGF1,2]. An exit four-week PET was obtained for comparison with the baseline scan.

## Results

Ten subjects with diverse cancers completed ≥ 26 days of INSINH without associated unsafe adverse effects. Mean caloric intake decreased (35 ± 6) % vs. predicted requirements despite our best efforts to encourage increased food consumption. Weight loss (median 4%, range 0.0-6.1%) was not judged a health risk in any subject. Mild, reversible side effects included constipation (n=2), transient fatigue (n=5), and leg cramps (n=2). Among nine patients with pre-trial progressive disease (PD) five demonstrated post-trial stable disease or partial remission (SD/ PR) on PET. SD/PR correlated with three-fold higher ketosis compared to those with continued PD (n=4), (p<0.02), but was uncorrelated with reduced calorie intake (p=0.45) or weight loss (p=0.81). Insulin correlated inversely with ketosis (r=0.62, p=0.026), but did not correlate with IFG(1 or 2).

## Conclusions

Preliminary pilot data in ten subjects demonstrated that an INSINH diet is safe and feasible in selected patients with advanced cancer. The extent of ketosis, but neither calorie deficit nor weight loss correlated with SD/PR. The small sample size requires further study. Further exploration is also required to evaluate an insulin inhibiting diet’s: 1. mechanism in relation to calorie restriction; 2. role as an adjunct to metabolic or cytotoxic therapies; 3. long-term value to reduce overall cancer risk.
